# Role of Maternal Body Composition and Associated Factors in the Nutritional Status of Under-Five Children of South India: Findings From the Comprehensive National Nutritional and Health Survey

**DOI:** 10.7759/cureus.95396

**Published:** 2025-10-25

**Authors:** Mahesh Kumar Mummadi, Jag Jeevan Babu Geddam, Karthikeyan Ramanujam, Sridevi Manchala, Raghavendra Pandurangi, Venkat Rajireddy Garlapati, Sreenu Pagidoju, Laxmaiah Avula

**Affiliations:** 1 Clinical Epidemiology Division, ICMR-National Institute of Nutrition, Hyderabad, IND; 2 Public Health Nutrition, ICMR-National Institute of Nutrition, Hyderabad, IND

**Keywords:** body composition, cnnhs, maternal, socioeconomic, undernutrition

## Abstract

Background: Insufficient dietary intake is a leading cause of undernutrition among children, leading to delayed growth, low weight, and low cognitive ability, leading to decreased potential and functional capacity. These effects are mostly irreversible. A cross-sectional study was conducted in Southern India to evaluate maternal body composition and socioeconomic factors, and to examine their impact on the nutritional status of children under five years.

Methods: A community-based study where 4166 individuals from 900 households were covered under CNNHS (Comprehensive National Nutrition and Health Survey) from 42 villages and 18 wards in a South Indian district. For the present study of 907 mothers and 996 children, socioeconomic, demographic, and anthropometric particulars were assessed for the risk of stunting, wasting, and underweight in children with maternal body composition indicators, where chi-square tests, logistic regression, and adjusted odds ratios were used.

Results: The prevalence of stunting, wasting, and underweight was 35.95%, 17.69%, and 31.7%, respectively, in under-five children. Underweight prevalence in children is associated with maternal body composition, like maternal height, weight, waist circumference, hip circumference, body mass index, and body fat percentage.

Conclusion: It is concluded that maternal and child health indicators, such as mean height, weight, and body composition metrics, were linked to the nutritional status of under-five children. The prevalence of stunting, wasting, and underweight varied by age group, with significant associations found between these conditions and socioeconomic factors like household income, sanitation, and maternal education. Maternal characteristics, including height, weight, and body mass index, were strongly associated with children’s nutritional outcomes. Logistic regression analysis also highlighted that lower maternal education, weight, and height, as well as inadequate household facilities, were also associated with the risk of stunting, wasting, and underweight in under-five children.

## Introduction

Healthy and balanced nutrition enables children to survive, thrive, and contribute to society, while undernutrition deprives them of their full potential and functional capacity. Undernutrition, resulting from inadequate nutrient intake, leads to delayed growth, underweight, and wasting [1], posing a major barrier to human development. Nutritional stunting is linked to brain structural and functional deficits and cognitive impairment. Wasting reflects acute malnutrition from insufficient food or frequent illness, whereas stunting signifies chronic undernutrition with largely irreversible effects. In 2020, an estimated 149 million children under five years were stunted, 45 million wasted, and 38.9 million overweight or obese globally, with southern Asia accounting for nearly two-fifths of stunted and over half of wasted children [2]. Among the wasted, one-third (14.3 million) were severely affected [3], emphasizing the urgent need for targeted actions to meet the 2030 Sustainable Development Goal on reducing child undernutrition. Key contributing factors include inadequate maternal nutrition, intrauterine undernutrition, lack of exclusive breastfeeding for the first six months, delayed or insufficient complementary feeding, and impaired nutrient absorption due to infections [4-6]. Undernourished children are more vulnerable to illness [3,7,8], show reduced cognitive and academic performance, have lower adult productivity, and face a higher risk of non-communicable diseases [6]. The intergenerational impact of poor nutrition begins in utero and persists across generations [9]. Undernourished women are more likely to have low-birthweight infants who experience suboptimal growth [10,11], and early childhood growth failure predicts adult stunting [12,13]. Addressing child undernutrition and stunting is therefore vital for lifelong health and human capital development.

Previous research indicates that triceps skinfold thickness is crucial for calculating upper arm muscle circumference, providing insights into body fat and protein reserves. Anthropometric measures such as waist and hip circumferences, along with skinfold thickness, are commonly used due to their simplicity and cost-effectiveness [14,15]. However, only a few studies have compared body mass index (BMI) with body fat percentage (%BF). In this study, skinfold thickness measurements were used to estimate %BF, considered a more accurate indicator of maternal nutritional status than BMI [16]. Measurements of biceps, triceps, subscapular, and suprailiac skinfolds offer valuable information on pregnancy-related adipose changes that are not influenced by fetal growth or edema [17]. Furthermore, mid-upper arm circumference (MUAC) is recognized as an effective indicator of maternal nutrition due to its strong correlation with body weight [18-21]. Therefore, this study aims to examine the association between maternal body composition, including detailed metrics such as skinfold-derived %BF, waist and hip circumference, and the nutritional status of children under five years of age, while accounting for household (HH) socioeconomic factors.

## Materials and methods

Study design and setting

A cross-sectional, community-based study, the Comprehensive National Nutritional and Health Survey (CNNHS), was carried out during 2018-19 in rural and urban areas, including 42 villages and 18 wards in Nalgonda district, Telangana state in South India, by adopting a multistage stratified random sampling procedure. The sample size for the study was estimated based on the existing prevalence of stunting in children under five years, i.e., 38%, at a 95% confidence interval, 6% absolute precision, 1.5 design effect, and 10% non-response. Thus, a sample size of 415 was arrived at, which needs to be covered in approximately 900 HH. Detailed methodology for sample size estimation is provided in Supplementary Table 4. The sample was selected based on the population proportionate to size (PPS) method, and from each selected village/ward, 15 HHs were selected.

Sample Population

The study encompassed 4,166 individuals from 900 HHs, with data collected on socioeconomic and demographic characteristics, anthropometry, and dietary intake. After applying the exclusion criteria of all the other age groups apart from mothers of under-five children and under-five children, 3,170 individuals were excluded. Among the remaining participants, 907 mothers and 996 children met the inclusion criteria. Due to missing anthropometric or maternal data, 89 children and 14 mothers were further excluded. As a result, complete data were available for 893 mothers and 907 children. To ensure equal mother-child pairing, the final analysis was conducted on 893 matched pairs.

**Figure 1:**
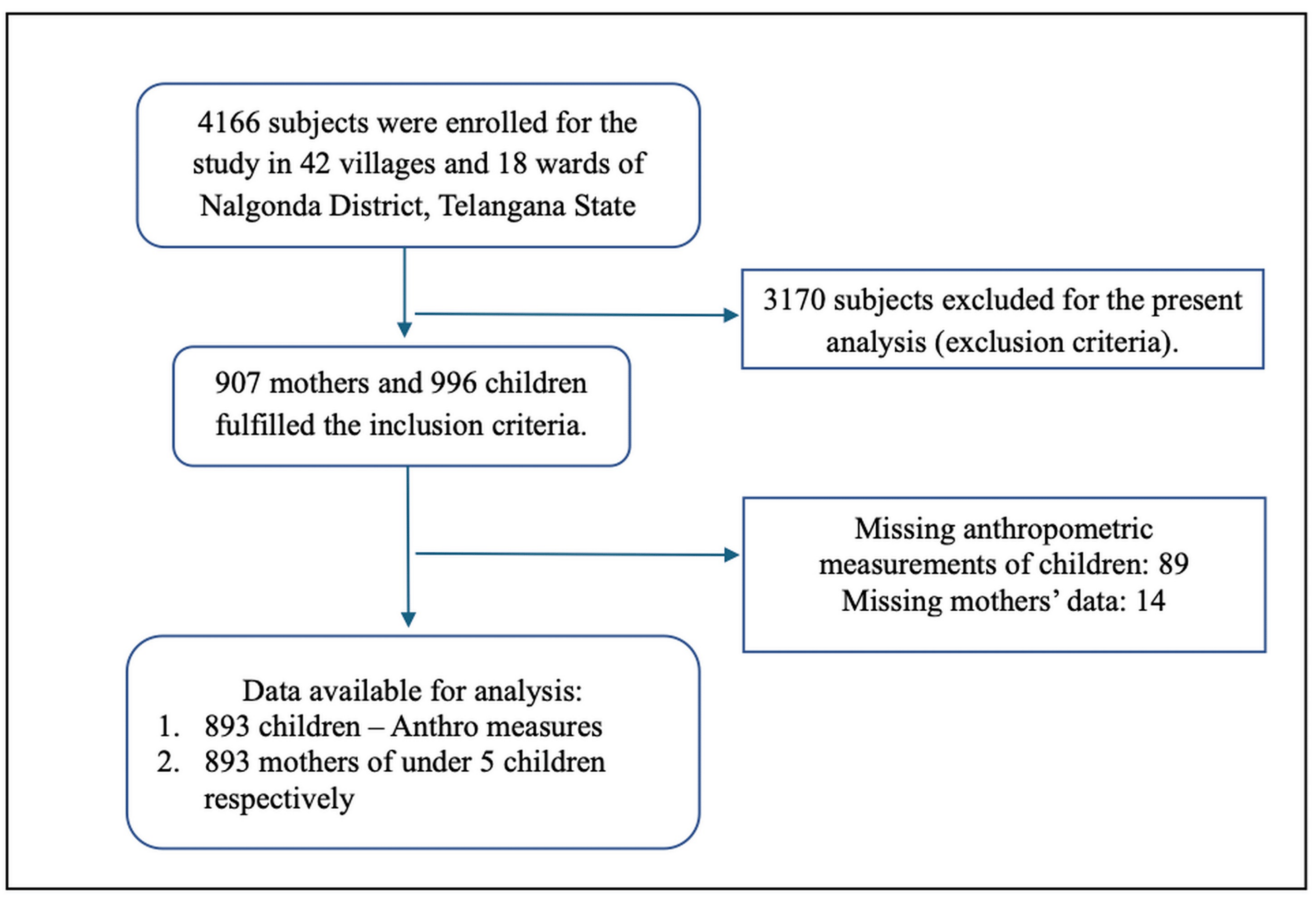
Flowchart of participant enrollment and final sample selection in the Nalgonda - CNNHS Study Out of 4166 subjects enrolled from 42 villages and 18 wards in Nalgonda District, Telangana, 3170 were excluded based on the exclusion criteria. Of the 907 mothers and 996 children meeting the inclusion criteria, data loss occurred due to missing anthropometric or maternal information. The final analysis included 893 mother–child pairs with complete data. CNNHS: Comprehensive National Nutrition and Health Survey.

The details of the study participants are presented as a flow diagram and are shown in Figure 1.

Inclusion criteria

The subjects included in this study were mothers and children under the age of five years, as this study involves HH characteristics, maternal indicators, and nutritional status of children under the age of five.

Exclusion criteria

The data about other age groups of children and adults are excluded from the present study as they are not included in this analysis.

Explanatory variables

1. Socioeconomic and Demographic Particulars

Data was collected using pre-tested structured questionnaires (The structured questionnaires were adapted from previously validated NFHS Questionnaire. Pre-testing was conducted in a demographically similar population to assess clarity and relevance, with modifications made based on field feedback. Socio-economic and demographic details, maternal indicators, knowledge on health practices during pregnancy, income particulars, and birth outcomes of children were collected, and the nutritional status of children was calculated.

2. Maternal Anthropometry

Mean maternal height, weight, MUAC, and fat-fold thickness (FFT) at biceps, triceps, and subscapular and suprailiac, and waist and hip circumference were measured with standard equipment (SECA) and procedures [22]. MUAC was measured in centimeters on the right arm, at the midpoint between the acromion and olecranon processes, to the nearest decimal place, and compared with the median values of WHO growth standards. Triceps, biceps, and subscapular and suprailiac skinfold thickness were measured by trained staff using a Lange skinfold caliper (nearest to 1 mm) following standardized methods. Measurements of waist and hip circumference were performed on the right side using a non-stretch measuring tape to the nearest 0.1 cm. Waist and hip circumferences were used to calculate the waist: hip ratio. The height and weight of all subjects were measured using a portable SECA scale (SECA robusta 813, Hamburg, Germany) to the nearest 0.1 kg and an SECA height rod to the nearest 0.1 cm (SECA 213 portable stadiometer). Following earlier studies, the measurements of biceps, triceps, and subscapular skinfold thickness, along with height, were used in the present study to estimate the %BF. The following formula [17] is used to estimate the %BF from the skin fold thickness measurements (SFTM): %BF = 12.5 + (0.457 × Triceps SFTM) + (0.352 × subscapular SFTM) + (0.103 × Biceps SFTM) - (0.057 × Height) + (0.265 × Arm) Circumference.

3. Nutritional Status of Children

The nutritional status of children was assessed based on the WHO Child Growth Standards [23], where HFA (height for age - stunting), WFH (weight for height - wasting), and WFA (weight for age - underweight) have been calculated. The nutritional status of mothers was assessed based on BMI [24] and classification suggested by the WHO Consultative group for Asians %BF: Subjects were classified based on earlier studies [25]: (BF%: low: <22.3 ± 5.1; medium: 24.4 ± 5.0; high: >26.6 ± 4.8).

Statistical analysis

Data were entered into MS Excel (Microsoft, Redmond, WA, USA) and analyzed using STATA 15 version (StataCorp., College Station, TX, USA). Descriptive statistics, chi-square tests, and logistic regression were computed to assess the risk of stunting, wasting, and underweight in children with various cutoff points of maternal indicators and socioeconomic characteristics of the HHs.

Logistic regression was performed between maternal anthropometric variables and key socioeconomic indicators with respect to stunting, wasting, and undernutrition status of under-five children. 

Adjusted odds ratios from multivariable regression models were also calculated to examine the association between maternal anthropometric factors and major socioeconomic factors with respect to stunting, wasting, and underweight of children under five years of age. 

Thus, mother's education, height, weight, BMI, MUAC, FFT scapular, waist and hip circumference, house ownership, presence of sanitary latrine, source of drinking water, cooking fuel, presence of separate kitchen, availing public distribution system, family size, and monthly per capita income are the variables listed in the final model as p < 0.05 in the earlier logistic regression.

Missing Data Handling

Subjects with missing anthropometric or maternal data were excluded from the final analysis. We performed a complete case analysis and did not apply imputation techniques.

## Results

Maternal and child indicators

The mean height and weight of the mothers were 152.3 cm (±6.19) and 50.66 kg (±11.09), respectively. MUAC was 24.62 cm (±4.36), FFT at triceps, biceps, subscapular, and suprailiac were 16.26 mm, 10.94 mm, 14.50 mm, and 11.51 mm, respectively. The mean waist circumference was 78.47 cm, the hip circumference was 86.93 cm, and the mean BMI was 22.09 kg/m^2^. Mean BF% was 25.3. The mean age of the children was 2.42 years (±1.27), and the mean weight and height of the children were 10.65 kg (±2.88) and 83.77 cm (±11.590), respectively. Of the total 893 children under the age of five, 429 (48.04%) were boys and 464 (51.96%) were girls.

Nutritional status of children under the age of five

The prevalence of moderate and severe stunting was 22.6% and 13.3%, respectively, while moderate and severe wasting was 11.4% and 6.27%, respectively. The prevalence of moderate and severe underweight was 22.06% and 9.6%, respectively, in the study area.

**Figure 2:**
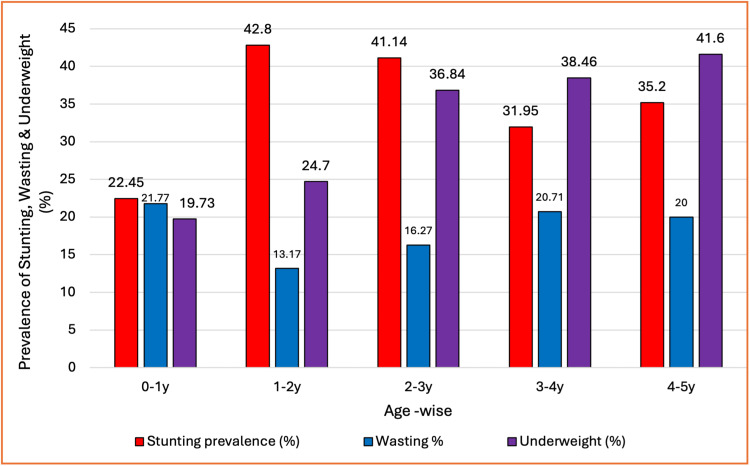
Prevalence of stunting, wasting, and underweight among children under five years The bar chart presents the age-specific distribution of undernutrition indicators among children under five years. Stunting prevalence peaks at 1-2 years (42.8%) and remains high at 2-3 years (41.1%). Wasting is most prevalent in infancy (0-1 year: 21.8%), while underweight steadily increases with age, reaching 41.6% at 4-5 years. These findings highlight critical age windows for targeted nutrition interventions. Stunting prevalence: height for age (<-2SD); wasting: weight for height (<-2SD); underweight: weight for age (<-2SD).

The prevalence of stunting is highest (42.8%) in the 1-2-year age group children, and lowest in the 0-1-year age group children (22.45%) as shown in Figure 2. The prevalence of wasting is the highest (21.7%) in the 0-1-year age group children and lowest (13.17%) in the 1-2-year age group children. The prevalence of underweight has been increasing steadily with age (41.6%), with the highest in the 4-5-year age group children.

Socioeconomic and demographic characteristics and nutritional status of children under the age of five

Table 1 provides a snapshot of the general demographic information of the study population. The sociodemographic characteristics of the HH are found to be strongly associated (p < 0.001) with stunting prevalence in children for sanitary latrine, type of fuel used for cooking, having a separate kitchen, and monthly per capita income. Among children with wasting, statistically significant associations were observed with residential area, house ownership, source of drinking water, and access to the public distribution system. In underweight children, religion, sanitary latrine, and cooking type were found to have a statistically significant association (p < 0.01), while separate kitchen, family size, and per capita income were found to have a statistically significant association (p < 0.05). There seemed to be no association between electricity and the type of house with the nutritional status of children under the age of five in the study area.

**Table 1:** Association between socioeconomic and demographic characteristics of the mothers and children under the age of five with nutritional status in the Nalgonda - CNNHS Study This table presents the baseline sociodemographic details of study participants, including mothers and their children under the age of five years. Variables reported include maternal age, education, occupation, socioeconomic status, and child-related factors such as age and sex distribution. These data provide context for interpreting nutritional and health outcomes assessed in the study. *p≤0.05; **p≤0.01; ***p≤0.001. CNNHS: Comprehensive National Nutrition and Health Survey; stunting: height for age (<-2SD); wasting: weight for height (<-2SD); underweight: weight for age (<-2SD).

Sociodemographic factors		No. of children	Stunting			Wasting			Underweight		
			N	%	p-Value	N	%	p-Value	N	%	p-Value
Area	Rural	628	229	36.5	0.619	101	16.1	0.052	201	32	0.755
	Urban	265	92	34.7		57	21.5		82	30.9	
Religion	Hindu	849	310	36.5	0.359	155	18.3	0.257	276	32.5	0.079
	Muslim	20	6	30		2	10		5	25	
	Christian	22	5	22.7		1	4.5		2	9.1	
	Others	2	0	0		0	0		0	0	
Type of House	Pucca	649	224	34.5	0.157	116	17.9	0.91	199	30.7	0.556
	Semi-Pucca	223	86	38.6		39	17.5		77	34.5	
	Kutcha	21	11	52.4		3	14.3		7	33.3	
House Ownership	Own	747	274	36.68	0.496	122	16.3	0.036*	232	31.1	0.152
	Rented	141	46	32.62		34	24.1		51	36.2	
	Living in others' house	5	1	20		2	40		0	0	
Type of Family	Nuclear	635	235	37	2.097	123	19.4	0.1	214	33.7	0.105
	Extended nuclear	223	77	34.5		29	13		58	26	
	Joint	35	9	25.7		6	17.1		11	31.4	
Sanitary Latrine	Latrine	699	232	33.2	0.001***	130	18.6	0.179	211	30.2	0.067
	Open defecation/not using latrine	194	89	45.9		28	14.4		72	37.1	
Source of Drinking Water	Draw well/Tube well	7	3	42.9	0.199	4	57.1	0.019*	3	42.9	0.108
	Tap water	475	183	38.5		79	16.6		164	34.5	
	Packed/Filtered water	411	135	32.8		75	18.2		116	28.2	
Electricity	Yes	876	317	36.2	0.281	156	17.8	0.518	276	31.5	0.396
	No	17	4	23.5		2	11.8		7	41.2	
Cooking Type	Firewood/Kerosene	64	35	54.7	0.001***	11	17.2	0.912	27	42.2	0.061
	LPG/Biogas	829	286	34.5		147	17.7		256	30.9	
Separate Kitchen	Yes	508	158	31.1	0.001***	85	16.7	0.387	145	28.5	0.02*
	No	385	163	42.3		73	19		138	35.8	
Public Distribution System (PDS)	Yes	810	292	36	0.841	136	16.8	0.027*	261	32.2	0.286
	No	83	29	34.9		22	26.5		22	26.5	
Family Size	<4	108	35	32.4	0.414	19	17.6	0.977	25	23.1	0.042*
	≥4	785	286	36.4		139	17.7		258	32.9	
Monthly Per Capita Income (Rs.)	>2500	278	123	44.2	0.001***	52	18.7	0.875	98	35.3	0.052
	2500-3890	346	117	33.8		57	16.5		111	32.1	
	>3890	269	81	30.1		49	18.2		74	27.5	
Iodine Content of Cooking Salt	0 ppm	121	41	33.9	0.073	17	14	0.558	35	28.9	0.315
	7 ppm	73	30	41.1		16	21.9		27	37	
	<15 ppm	486	188	38.7		88	18.1		162	33.3	
	≥15ppm	213	62	29.1		37	17.4		59	27.7	
Household Food Insecurity Score	0	877	131	14.9	0.237	153	17.4	0.152	276	31.5	0.295
	≥1	16	8	50		5	31.3		7	43.8	
Number of Assets	≤6	709	262	37	0.218	118	16.6	0.107	220	31	0.404
	>6	184	59	32.1		40	21.7		63	34.2	

Maternal indicators, body composition, and nutritional status of children under the age of five

Our study finds that stunting prevalence in children is strongly associated with mother’s height and weight (p = 0.001); statistically significantly associated with mother’s MUAC, FFT, waist circumference, and %BF (p < 0.05); and significantly associated with maternal BMI and hip circumference (p < 0.01) (Table 2). Wasting prevalence in children is strongly associated with mothers’ weight and hip circumference (p = 0.001), significantly associated with mother’s height, MUAC, BMI, waist circumference, FFT, and %BF (p < 0.05), and statistically associated with mother’s education (p < 0.01). Underweight prevalence in children is strongly associated with maternal height, weight, waist circumference, and hip circumference (p < 0.001), statistically significantly associated with maternal BMI, MUAC, FFT, and %BF (p < 0.05), and significantly associated with mother’s education and occupation (p < 0.01).

**Table 2:** Association between maternal body composition and other indicators with nutritional status of children under the age of five in Nalgonda - CNNHS Study The table summarizes the prevalence of undernutrition among children under five years across different age groups with respect to maternal body composition. Indicators include stunting, wasting, and underweight, expressed as percentages. The data highlight age-related variations in nutritional status, with stunting peaking at 1-2 years and underweight showing a progressive increase with age. *p≤0.05; **p≤0.01; ***p≤0.001. BMI: body mass index; MUAC: mid-upper arm circumference; FFT: fat-fold thickness; stunting: height for age (<-2SD); wasting: weight for height (<-2SD); underweight: weight for age (<-2SD); CED: chronic energy deficiency.

Maternal indicators		Total children	Stunting			Wasting			Underweight		
			N	%	p-Value	N	%	p-Value	N	%	p-Value
Mother's Education	Illiterate	91	36	39.6	0.023*	12	13.2	0.084	34	37.4	0.091
	Read & write	50	21	42		9	18		17	34	
	1-4 standard	31	12	38.7		4	12.9		7	22.6	
	5-8 standard	122	47	38.5		15	12.3		45	36.9	
	9-12 standard	422	157	37.2		80	19		129	30.6	
	Degree/PG	177	48	27.1		38	21.5		51	28.8	
Mother's Occupation	Labourer	80	34	42.5	0.253	13	16.3	0.932	34	42.5	0.085
	Cultivators/Service/Business	143	45	31.5		25	17.5		47	32.9	
	Housewife/Dependents	670	242	36.1		120	17.9		202	30.1	
Mother's Weight (kg)		445	185	41.6	0.001***	97	21.8	0.002**	169	38	<0.001***
	≥Median	443	136	30.7		61	13.8		113	25.5	
Mother's Height (cm)		437	186	42.6	<0.001***	91	20.8	0.02*	164	37.5	<0.001***
	≥Median	451	135	29.9		67	14.9		118	26.2	
Maternal BMI (WHO) (kg/m^2^)	CED (<18.5)	182	77	42.3	0.23	44	24.2	0.053	74	40.7	0.031*
	Normal (18.5-24.9)	524	184	35.1		88	16.8		155	29.6	
	Overweight (25-29.9)	146	49	33.6		23	15.8		45	30.8	
	Obese (≥30)	35	10	28.6		3	8.6		8	22.9	
Maternal BMI (Asian) (kg/m^2^)	CED (<18.5)	182	77	42.3	0.09	44	24.2	0.031*	74	40.7	0.034*
	Normal (18.5-22.99)	407	148	36.4		74	18.2		124	30.5	
	Overweight (23-27.49)	215	65	30.2		29	13.5		60	27.9	
	Obese (≥27.5)	82	30	36.6		11	13.4		24	29.3	
Maternal Age (years)		446	165	37	0.514	74	16.6	0.389	131	29.4	0.137
	≥Median	447	156	34.9		84	18.8		152	34	
Mother's MUAC (cm)		445	159	35.7	0.01**	175	39.3	0.036*	93	20.9	0.012*
	>Median	448	124	27.7		146	32.6		65	14.5	
Maternal FFT Triceps (mm)		445	142	31.9	0.888	169	38.0	0.207	76	17.1	0.632
	>Median	448	141	31.5		152	33.9		82	18.3	
Maternal FFT Biceps (mm)		438	138	31.5	0.91	161	36.8	0.673	80	18.3	0.694
	>Median	452	144	31.9		160	35.4		78	17.3	
Maternal FFT Scapular (mm)		428	151	35.3	0.035*	171	40.0	0.027*	88	20.6	0.042*
	>Median	457	131	28.7		150	32.8		70	15.3	
Maternal FFT Suprailiac (mm)		442	144	32.6	0.665	170	38.5	0.184	81	18.3	0.725
	>Median	442	138	31.2		151	34.2		77	17.4	
Maternal Waist Circumference (cm)		440	156	35.5	0.023*	176	40.0	0.022*	96	21.8	0.002**
	>Median	445	126	28.3		145	32.6		62	13.9	
Maternal Hip Circumference (cm)		442	154	34.8	0.058	181	41.0	0.004**	94	21.3	0.008**
	>Median	443	128	28.9		140	31.6		64	14.4	
% Body Fat	Low (22.3 ± 5.1)	350	145	41.4	0.026*	74	21.1	0.045*	124	35.4	0.031*
	Medium (24.4-26.5 ± 5)	116	36	31.0		13	11.2		26	7.4	
	High (>26.6 ± 4.8)	409	135	33.0		70	17.1		127	36.3	

Regression analysis

Logistic regression analysis found mothers’ ability to read and write, low weight and height, HHs not having a separate kitchen, and lower maternal hip circumference as higher risk for stunting in children under the age of five. There also seems to be significant association of mothers having lower education, height, weight, MUAC, waist circumference, living in rented house, living in others house, and not having access to public distribution system with higher risk for wasting in children under the age of five, while there seems to be an association of lower maternal weight, lower height, low BMI or CED of mothers, and not having separate kitchen with higher risk for underweight children (Table 3). It can be observed that maternal education is not associated with underweight prevalence in children. However, the odds of stunting in children decrease with an increase in the education of mothers.

**Table 3:** Regression analysis for predictors of stunting, wasting and underweight prevalence in children under the age of five in Nalgonda - CNNHS Study This table presents the AORs with 95% confidence intervals for the relationship between maternal characteristics (education, anthropometry, BMI, MUAC, waist–hip measures) and household factors (sanitation, water source, cooking type, income, etc.) with childhood stunting, wasting, and underweight. Significant associations were observed for maternal weight, maternal height, and household water source, as well as income levels, indicating their influence on child nutritional outcomes. *p≤0.05; **p≤0.01; ***p≤0.001. BMI: body mass index; MUAC: mid-upper arm circumference; FFT: fat-fold thickness; Stunting: height for age (<-2SD); wasting: weight for height (<-2SD); underweight: weight for age (<-2SD); CED: chronic energy deficiency; AOR: adjusted odds ratio.

Maternal anthropometric and socioeconomic characteristics	Categories	Stunting			Wasting			Underweight			
											AOR	95% CI	p-Value	AOR	95% CI	p-Value	AOR	95% CI	p-Value	
		Mother's Education	Illiterate	1	-	-	1	-	-				
Read & write	1.15		0.54-2.41	0.712	1.34	0.50-3.55	0.555				
1-4 standard	1.03		0.43-2.50	0.935	0.79	0.22-2.87	0.732				
5-8 standard	0.85		0.47-1.52	0.589	0.76	0.33-1.76	0.527				
9-12 standard	1.02		0.62-1.69	0.922	1.32	0.67-2.62	0.418				
Degree/PG	0.744		0.41-1.34	0.327	1.49	0.70-3.14	0.293				
Mother's Weight (kg)		1.64	1.22-2.20		1.12	0.60-2.08	0.719	1.64	1.03-2.63	0.037*	
	≥Median										
Mother's Height (cm)		1.25	0.80-1.94		1.52	1.03-2.24	0.031*	1.56	1.16-2.11	0.003**	
	≥Median										
Maternal BMI (WHO) (kg/m^2^)	Normal (18.5-24.9)				1			1			
	CED (<18.5)				1.09	0.66-1.81	0.719	1.24	0.81-1.91	0.309	
	Overweight (25-29.9)				0.84	0.42-1.63	0.603	1.4	0.85-2.29	0.175	
	Obese (≥30)				0.93	0.39-2.21	0.869	1.05	0.43-2.54	0.903	
Mother's MUAC (mm)		1.01	0.66-1.54	0.958	1.16	0.63-2.02	0.58	1.08	0.69-1.70	0.72	
	>Median										
Maternal FFT scapular (mm)		1.03	0.70-1.52	0.872	1.07	0.66-1.74	0.77	1.03	0.69-1.55	0.852	
	>Median										
Maternal Waist Circumference (cm)		1.01	0.68-1.49	0.956	1.32	0.80-2.19	0.263	1.04	0.69-1.58	0.835	
	>Median										
Maternal Hip Circumference (cm)		1.19	0.80-1.77	0.386	0.97	0.58-1.62	0.927	0.85	0.56-1.28	0.835	
	>Median										
House Ownership	Own				1						
	Rented				1.5	0.93-2.41	0.093				
	Living in Others' houses				3.74	0.55-25.3	0.175				
Sanitary Latrine	Latrine	1									
	Open defecation/not using a latrine	1.26	0.87-1.84								
Source of Drinking Water	Draw well/Tube well				1	-	-				
	Tap water				0.19	0.04-0.93	0.041*				
	Packed/Filtered water				0.2	0.42-1.02	0.053				
Cooking Type	Firewood/Kerosene	1									
	LPG/Biogas	0.69	0.38-1.23								
Separate Kitchen	No	1.27	0.93-1.72					1.26	0.93-1.70	0.133	
	Yes										
Public Distribution System (PDS)	Yes				1						
	No				1.7	0.98-2.94	0.057				
Family Size	<4							0.624	0.38-1.02	0.065	
	≥4										
Monthly Per Capita Income	<2500	1						1	-	-	
	2500-3890	0.68	0.48-0.96	0.031*				0.95	0.67-1.35	0.809	
	>3890	0.69	0.47-1.01	0.063				0.84	0.56-1.25	0.398	

## Discussion

The prevalence of stunting, wasting, and underweight was 35.95%, 17.69%, and 31.7%, respectively, in the study area when compared to the prevalence rates in Telangana state at 33.1%, 21.7%, and 31.8%, respectively. While at the national level, the prevalence of stunting, wasting, and underweight was 35.5%, 19.3%, and 32.1%, respectively. This study finds that the prevalence of stunting is the highest in the 2-3-year age group children and lowest in infants, while wasting is highest among infants. The prevalence of underweight increased with age and is the highest in the 4-5-year age group children [26].

Evidence shows that socioeconomic and demographic factors are key contributors to inadequate nutrition among mothers and children. Our analyses on the nutritional status in children under the age of five show lower prevalence of stunting and underweight in respondents with pucca houses, and lower prevalence of wasting and underweight in children is visible in respondents with their own house, while, on the contrary, stunting is higher in children belonging to respondents with their own house. The prevalence of stunting, wasting, and underweight is higher in children belonging to nuclear families when compared to other joint families, attributing to a higher number of working members in a joint family. The prevalence of stunting and underweight prevalence in children is lower in HHs having a latrine, in contrast to wasting prevalence in children in HHs with a latrine, attributing to other contributing factors.

The variations in sociodemographic characteristics of the HHs and the nutritional status of children in the study area show that underweight and stunting prevalence are higher in rural over urban areas, while wasting prevalence is lower in rural areas and vice versa. Higher prevalence of stunting, wasting, and underweight is evident in Hindus when compared to all other religions. Stunting and underweight prevalence are lower in those living in pucca houses, in contrast to the higher prevalence of wasting in pucca houses. Underweight and wasting prevalence is found to be lower in those living in owned houses, while stunting is higher. 

It is surprising to note that stunting, wasting, and underweight prevalence are lower in joint and extended nuclear families, in HHs using LPG for cooking, and in HHs having a separate kitchen. Stunting and underweight prevalence are lower in HHs with sanitary latrine, and in HHs having filtered water, while wasting is lower in HHs having tap water. It is observed that stunting, wasting, and underweight in children are associated with mothers' education, height, weight, and BMI (Asian classification), while mothers' occupation is associated with underweight prevalence in children with statistical significance p < 0.01 (p = 0.085). It can be observed that maternal age is not associated with stunting, wasting, or underweight prevalence in children. 

Earlier studies revealed that the odds of stunting reduced by 4% with a unit increase in maternal BMI in Ethiopia. A similar analysis in India reported that stunting reduced by 3% with a unit increase in the BMI of mothers [27]. Here, our study has included skinfold thickness measures to calculate the %BF, which is considered a better measure to estimate the nutritional status in mothers when compared to BMI, as revealed by an earlier study [16]. Prior studies support %BF as a better predictor of fetal growth and infant adiposity, reinforcing its relevance beyond birth outcomes. However, this study highlights a novel association between maternal body composition, particularly %BF derived from skinfold thickness, and child undernutrition.

This study underscores the link between maternal indicators, particularly body composition, and the nutritional outcomes of children below five years of age. Stunting is found to be strongly associated with mothers' height and weight, FFT subscapular, waist circumference, %BF, hip circumference, BMI (Asian classification), and MUAC. The prevalence of wasting is strongly associated with mothers' weight and hip circumference, height, BMI, MUAC, FFT scapular, waist circumference, %BF, and education. The prevalence of underweight is strongly associated with maternal hip circumference, waist circumference, weight and height, BMI, FFT scapular, MUAC, %BF, occupation, and education. Several studies revealed the association between SFTM on perinatal outcomes with different conclusions. A 1997 study [28] showed that maternal arm fat was correlated to infant %BF, and maternal arm muscle area was related to infant length or height. While changes in maternal arm fat and arm circumference during gestation were correlated with infant birth weight in teenage pregnancies in 1988 study [29], maternal SFTM during the second trimester was inversely associated with infant birth weight, after adjustment for maternal age, BMI, and parity in a 2021 study [30]. Our study demonstrates that maternal body composition is strongly associated with the nutritional status of children, emphasizing the need for improvement in the body composition of mothers during pregnancy and lactation for better birth and nutrition outcomes. The assessment of %BF gives a better picture of the association of the body composition of the mothers with the nutritional status of the children in the present study.

A key strength of this study is the availability of a representative cross-sectional sample, with data on anthropometrics, body composition of the mothers comprising SFTM, through which %BF has been estimated as opposed to BMI, which is an indicator of obesity. The new insight of this study rests on the association of maternal body composition and maternal indicators with the nutritional status of children under the age of five. This study reinforces the importance of maternal nutritional status during pregnancy and lactation for ensuring better pregnancy and nutrition outcomes for the mother and child.

Highlighting the study’s limitations can pave the way for future research efforts. This study is cross-sectional and restricted to one district in Telangana state, and the findings of this study may not be generalized. It remains unclear whether poor maternal body composition leads to child undernutrition or whether shared socioeconomic stressors, such as large family size or inadequate resources, contribute to both. A few variables, like dietary intake, birth weight, and illness history, are not included due to the focus on only maternal body composition. Longitudinal research is needed to disentangle these pathways. Despite the limitations, this study makes an attempt to contribute to the limited data available on maternal body composition and socioeconomic indicators associated with the nutritional status of children in the study area for making better health policies.

## Conclusions

The study found that, considering the South Indian population, the maternal and child health indicators, such as mean height, weight, and body composition metrics, were linked to the nutritional status of children under the age of five. The prevalence of stunting, wasting, and underweight varied by age group, with significant associations found between these conditions and socioeconomic factors like HH income, sanitation, and maternal education. Maternal characteristics, including height, weight, and BMI, were strongly correlated with children’s nutritional outcomes. Logistic regression analysis highlighted that lower maternal education, weight, and height, as well as inadequate HH facilities, increased the risk of stunting, wasting, and underweight in children under the age of five.

## Appendices

**Table 4:** Detailed methodology for sample size estimation for each investigation MUAC: mid-upper arm circumference; FFT: fat-fold thickness; HH: household; IYCF: infant and young child feeding; TSH: thyroid-stimulating hormone; TC: total cholesterol; TG: triglycerides; LDL: low-density lipoprotein; HDL: high-density lipoprotein; SBP: systolic blood pressure; DBP: diastolic blood pressure; BIA: bioelectrical impedance analysis; WC: waist circumference; HC: hip circumference; HTN: hypertension; DM: diabetes mellitus.

Investigations	Age/Sex/Physiological group	Prevalence	CI	Absolute precision	Design effect	Non-response	Sample required per district/group
Sociodemographic particulars	All the selected HHs for different investigations will be covered			-	-	-	900HHs
	Under five years - Stunting	38%	95%	6%	1.5	10%	415
Anthropometry (height, weight, MUAC, FFT at triceps)for all the ages in the selected HH	All the available subjects in the selected households						2800 approx.
Clinical examination for nutritional deficiency signs and current morbidity	All available subjects in the selected households						2800 approx.
Diet surveys	One-third HHs selected for various investigations						300 HHs
IYCF practices	All available mothers of <5-year children covered for anthro						
Haemoglobin	6-59 months and 6-17 years and men and women ≥18 years	50%	95%	8%	1.5	10%	248
Micronutrients estimation: ferritin, serum transferrin receptor, CRP, zinc, vitamin A, B12, red blood folate, methyl-malonic acid, homocysteine, serum 25 (OH) D, serum protein and albumin, TSH	Children (6-17 years) and men ≥18 years	20%	95%	4%	1.5	10%	634
	Children (6-17 years) and women ≥18 years	20%	95%	4%	1.5	10%	634
Urinary iodine, sodium, creatine, and potassium concentration	Children (12-17 years) and men ≥18 years	20%	95%	4%	1.5	10%	634
	Children (12-17 years) and women ≥18 years	20%	95%	4%	1.5	10%	634
Diabetes	Children (12-17 years) and men ≥18 years	15%	95%	3%	1.5	10%	898
	Children (12-17 years) and women ≥18 years	15%	95%	3%	1.5	10%	898
Dyslipidaemia (TC, TG, HDL, LDL)	Children (12-17 years) and men ≥18 years	30%	95%	6%	1.5	10%	370
	Children (12-17 yeas) and women ≥18 years	30%	95%	6%	1.5	10%	370
Blood pressure measurement (SBP, DBP)	Children (12-17 years) and men ≥18 years	20%	95%	4%	1.5	10%	634
	Children (12-17 years) and women ≥18 years	20%	95%	4%	1.5	10%	634
Body composition (BIA, WC, HC, fat fold thickness multiple sites) adolescents and adults	Children (12-17 years) and men ≥18 years	20%	95%	4%	1.5	10%	634
	Children (12-17 years) and women ≥18 years	20%	95%	4%	1.5	10%	634
Assessment of 24-hour physical activity	Children (6-17 years) and men ≥18 years	-	-	-	-	-	634
	Children (6-17 years) and women ≥18 years	-	-	-	-	-	634
Knowledge and practice on HTN and DM	Children (6-17 years) and men ≥18 years	-	-	-	-	-	634
	Children (6-17 years) and women of ≥18 years	-	-	-			634
Water quality for mineral and trace elements	5 samples from each village/ward	-	-	-	-	-	300
